# PReS-FINAL-2332: Activation-induced cell death of human monocytes as a novel mechanism fine-tuning inflammation and autoimmunity

**DOI:** 10.1186/1546-0096-11-S2-P322

**Published:** 2013-12-05

**Authors:** M Miranda-García, J Däbritz, G Varga, T Weinhage, J Ehrchen, K Barczyk, J Roth, D Foell

**Affiliations:** 1 Pediatric Rheumatology and Immunology, University Hospital Muenster, Muenster, Germany; 2 Dermatology, University Hospital Muenster, Muenster, Germany; 3 Institute of Immunology, University Hospital Muenster, Muenster, Germany

## Introduction

Monocytes represent essential components of innate immunity with high plasticity.These cells are characterized by their ability to release large amounts of proinflammatory cytokines, efficient antigen presentation, and microbicidal or tumoricidal activity.They contribute to inflammation in the immune system, either by governing host defense response to invading pathogens or driving reactions to self-molecules in conditions of tissue-damage. Control of these mechanisms is necessary to ensure the self-limitation of inflammatory reactions and avoid perpetuated autoinflammation or autoimmunity. In T-cells activation-induced cell death is an important mechanism for maintaining tolerance to self-antigen. However, to date it is unclear how activated monocytes can regulate early cytokine signals promoting their survival or cell death.

## Objectives

In the light of pleiotropic functions and fundamental role of monocyte activation during early phases of inflammatory responses, we recapitulated activities of human monocytes in response to key Th17/Th1 cytokines that primarily affect monocytes (GM-CSF and IFNg).

## Methods

Primary human monocytes were isolated and subjected to stimulation with GM-CSF and IFNg. Cell death was measured using Annexin V and propidium-iodide staining and analyzed by FACS. Monocytes signaling pathways were analyzed by Western blot using antibodies against phosphorylated and non-phosphorylated proteins.TNF-blockers such as anti-TNF and etanercept were used to analyze the role of TNF in monocyte activation.

## Results

In the present study we demonstrate in vitro, that simultaneous treatment with GM-CSF and IFNg promotes activation-induced cell death (AICD) of human monocytes. Analyzing the signaling pathways that lead to cell death revealed that a specific mechanism described as pyronecrosis is induced by GM-CSF and IFNg. Pyronecrosis has morphological characteristics of necrosis, is caspase- and RIP kinase1- independent but cathepsin-B-dependent. GM-CSF/IFNg-induced cell death of monocytes involved IL-1b and TNFa-hypersecretion. Furthermore, pyronecrosis was found to be dependent on TNFa and could specifically be inhibited by TNF-blockers.

## Conclusion

Taken together, we identified AICD of monocytes as a novel mechanism, which could regulate inflammatory processes that may be altered in the context of autoinflammation or immunity. The involvement of different mediators and pathways in this process could have consequences on therapeutic strategies, e.g. for combination therapies involving TNF-blockers.

## Disclosure of interest

None declared.

